# Simultaneous Determination of 5 Components in the Leaves of *Dimocarpus longan* by Quantitative Analysis of Multicomponents by Single Marker (QAMS) Based on UPLC and HPLC

**DOI:** 10.1155/2020/3950609

**Published:** 2020-01-29

**Authors:** Jiani Mai, Jie Liang, XianFu Liu, LiuPing Tan, Hui Xu, YaoHua Li, YuShan Zhou, ChuanChuan Yang, ChenXi Xin

**Affiliations:** ^1^College of Pharmacy, Guangxi University of Chinese Medicine, Nanning 530222, Guangxi, China; ^2^GuangXi Key Laboratory of Zhuang and Yao Ethnic Medicine, Nanning 530200, China; ^3^Guangxi Zhuang Yao Medicine Center of Engineering and Technology, Nanning 530200, China; ^4^Faculty of Chinese Medicine Science, Guangxi University of Chinese Medicine, Nanning 530222, Guangxi, China

## Abstract

The pharmacodynamic effect of *longan* leaves was attributed to various components, especially the flavonoids. In this paper, a new strategy of quantitative analysis of multicomponents by a single marker (QAMS) method was first established to synchronously determine 5 components (ethyl gallate (C_1_), astragalin (C_2_), quercetin (C_3_), luteolin (C_4_), and kaempferol (C_5_)) in *Dimocarpus longan* by ultra-performance liquid chromatography (UPLC) and high-performance liquid chromatography (HPLC). Quercetin (C_3_) was chosen as the internal reference. Relative correction factors (RCF_s_, ƒ_s/i_) of the other 4 components were calculated by two correction methods (multipoint correction and slope correction) to effectuate QAMS. At the same time, the difference between the results measured by the QAMS and external standard methods was compared to verify the accuracy of QAMS. Within the linear range, the results showed that all ƒ_s/i_ values were obtained with good durability under diverse chromatographic conditions (RSD < 2.28%). The quantitative results of 5 components in the leaves of *Dimocarpus longan* collected from 10 producing areas by different chromatographic systems and quantitative methods were significantly correlated (Pearson's *r* > 97.0%). The applicability and feasibility of the QAMS method established in this study were evaluated to be favorable for quality control of the leaves of *Dimocarpus longan*. As a new model of quality control, it can provide one more choice of multicomponent quality-control method in the absence of standard substances or instruments.

## 1. Introduction

Quantitative analysis of multicomponents by a single marker (QAMS) is a new mode of multi-index evaluation. Using the relative correction factors between internal reference and other components, it only has to determine the internal reference in order to synchronously monitor the rest of the components [1–3]. Currently, the QAMS method has been used successfully in the quality control of various natural plant medicines in many countries [4]. For example, it has been collected in the China Pharmacopoeia (the 2015 Edition) to evaluate the quality of *Coptis chinensis* [5]. *Trifolium pratense*, *Hypericum perforatum*, *Ranunculus ternatus*, and *Rubus idaeus* extracts were recorded in the United States Pharmacopoeia (the 37 version). *Echinacea pallida* in the European Pharmacopoeia (EP 8.0) adopted QAMS [6–8].

Longan (*Dimocarpus longan* Lour.) is widely grown in Southern China especially in Guangxi, Guangdong, and Fujian provinces. In the antique book “*Herbal Medicines of Southern Yunnan*,” there was recordation about the leaf of *Dimocarpus longan* Lour having effective Chinese herbal medicine which can be used to treat cold, fever, malaria, malignant sore, and eczema [9].

The effect of different polar extracts from *Dimocarpus longan* leaves on regulating blood glucose in type 2 diabetic mice in different extent were published in our group [10, 11]. By Grey relational analysis and Pearson correlation analysis, it was found that the chromatographic peaks from *Dimocarpus longan* leaves were closely related to antioxidation and the activity of inhibiting *α*-glucosidase enriched in ethyl acetate extracts. At present, the main chemical constituents ethyl gallate (C_1_), astragalin (C_2_), quercetin (C_3_), luteolin (C_4_), and kaempferol (C_5_) have been found and separated from the leaves of *Dimocarpus longan* [12]. Studies have shown that C_1_, C_2_, C_3_, C_4_, and C_5_ in the leaves of *Dimocarpus longan* are the main components that exert pharmacological activity [13]. Therefore, it is meaningful to establish a quality standard that can rapidly and simultaneously evaluate the amount of these five components.

In our work, a new strategy of quantitative analysis of multicomponents by a single marker (QAMS) method for simultaneous quantification of 5 components in *Dimocarpus longan* by UPLC and HPLC was developed. Quercetin (C_3_) as an internal reference for its stable property, low price, and easily acquiring standard substance was used to calculate RCFs of the other 4 components. The QAMS method was first established to control the quality of *Dimocarpus longan* leaves more conveniently, comprehensively, and synthetically, meanwhile investigating the feasibility of UPLC and HPLC methods based on chromatographic condition transformation.

## 2. Materials and Methods

### 2.1. Chemicals and Reagents

**Figure 1:**
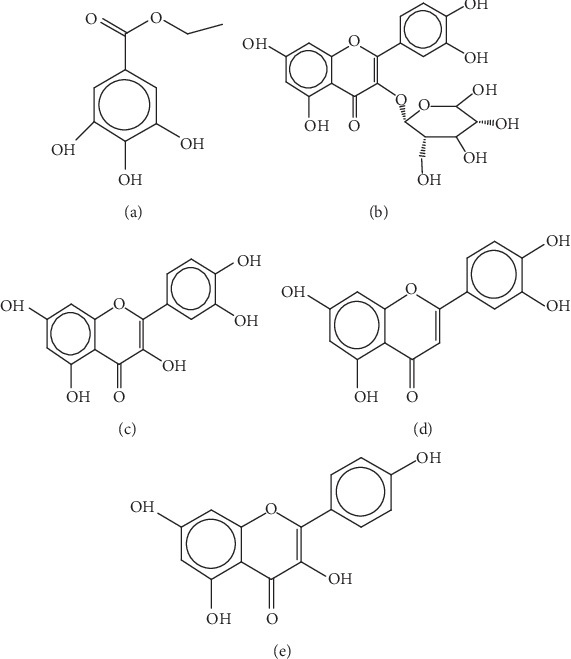
The structures of 5 marker constituents. (a) C_1_: ethyl gallate. (b) C_2_: astragalin. (c) C_3_: quercetin. (d) C_4_: luteolin. (e) C_5_: kaempferol.

Reference standard of C_3_ was purchased from National Institutes for Food and Drug Control (China). The other four standards of reference substance were obtained from Shanghai Winherb Medical Science Co., Ltd. (Shanghai, China). The purity of all standards was verified to be more than 99% and had laboratory accreditation certificate. The structures of 5 marker constituents are listed in Figure 1. Methanol (Dikma technology Co., Ltd., HPLC-grade) and ultrapure water purified with Millipore Simplicity. All other chemicals were AR grade.

### 2.2. Plant Materials

Leaves of *Dimocarpus longan* Lour. used in the experiment were collected from 10 regions in Guangxi province in China and authenticated by associate professor Jian-bei Teng from the Guangxi University of Traditional Chinese Medicine.

### 2.3. Instrument and Chromatographic Conditions

In the initial study, four solvents including methanol, ethanol, mixed solvent 1 (methanol : hydrochloric acid), and mixed solvent 2 (ethanol : hydrochloric acid) were adopted to extract the medicinal materials, and gradient elution procedures of four mobile phase systems including acetonitrile : 0.2% phosphoric acid, acetonitrile : water, methanol : water, and methanol : 0.2% phosphoric acid were also compared to help acquiring a more optimized approach. The results showed that mixed solvent 2 was the best solvent for extraction, and when methanol : 0.2% phosphoric acid was used as mobile-phase gradient eluent, the separation effect of each chromatographic peak tested was the best.

**Figure 2:**
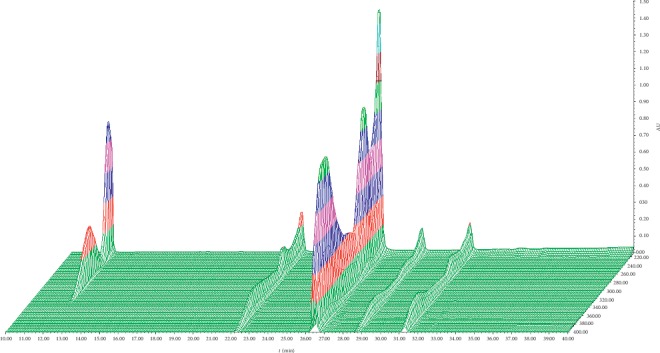
Full spectrum scan of five compounds.

The wavelength scanning of the sample set from 200 nm to 400 nm (Figure 2) showed that the maximum absorption wavelength of C_1_ was at about 280 nm, while the rest of the components were strongly absorbed at 360 nm. Therefore, the detection wavelength was changed after the peak of C_1_ in this study.

Analyses were performed on Agilent 1290 Infinity II and Agilent 1100 chromatographic systems equipped with a column temperature controller and VWD detector (Agilent, USA), respectively. The chromatographic separation was carried out on the reverse-phrase C_18_ columns including Thermo Syncronis C_18_ column (2.1ludi·mm, 1.7 *µ*m) and Waters ACQUITY UPLC HSS C_18_ column (2.1rs A·mm, 1.8 *µ*m). In the UPLC system, sample injection volume was 0.5 *µ*L. The mobile phase comprising methanol (A) and 0.2% phosphoric acid (B) was programmed with gradient elution (0–3 min, 20% to 30% A; 3–5 min, 30% to 38% A; 5–20 min, 38% to 75% A) at a flow rate of 0.2 mL/min. The column temperature was maintained at 30°C and detection wavelength was set at 280 nm and then changed to 360 nm after 10 min. According to the transformation formulas (1)–(3) [14, 15] and combining the actual situation after fine-tuning, the chromatographic conditions in HPLC determination can be intended as follows: Thermo Syncronis C_18_ column (2.1ermo·mm, 1.7 *µ*m) and Waters ACQUITY UPLC HSS C_18_ column (2.1rs A mm, 1.8 *µ*m) were used for separation. Sample injection volume was 5.0 *µ*L. The mobile phase was set with gradient elution (0–7 min, 20% to 30% A; 7–12 min, 30% to 38% A; 12–48 min, 38% to 75% A) at a flow rate of 1.0 mL/min. The column temperature was at 30°C and detection wavelength was set at 280 nm and then converted to 360 nm after 22 min.

(1) $$\begin{array}{c} \nu_{\text{target}}=\frac{\nu_{\text{original}}\cdot d_{\text{target}}^{2}}{d_{\text{original}}^{2}}, \end{array}$$

(2) $$\begin{array}{c} V_{\text{target injection}}=\frac{V_{\text{original injection}}\cdot V_{\text{target injection}}}{V_{\text{original column}}}, \end{array}$$

(3) $$\begin{array}{c} t_{\text{target}}=\frac{t_{\text{original}}\cdot \nu_{\text{original}}\cdot V_{\text{target column}}}{V_{\text{original column}}\cdot \nu_{\text{original}}}, \end{array}$$

where *ν* is the flow rate, *V* is the volume, *d* is the column inner diameter, and *t* represents the gradient time.

### 2.4. Preparation of Standard Solutions

The applicable amounts of C_1_, C_2_, C_3_, C_4_, and C_5_ were accurately weighed and then put into 5 mL volumetric flasks in methanol separately to make the stock solutions. The concentrations were as follows: C_1_, 1.226 mg/mL; C_2_, 0.270 mg/mL; C_3_, 4.160 mg/mL; C_4_, 0.230 mg/mL; C_5_, 0.722 mg/mL.

Working solution of mixtures of five standards was prepared by diluting the stock solutions to the concentration which contained 245.2 *µ*g C_1_, 108.0 *µ*g C_2_, 416.0 *µ*g C_3_, 46.0 *µ*g C_4_, and 72.2 *µ*g C_5_ per milliliter erewhile before analyses. Stocking solutions and working solutions were kept in dark and stored at 4°C.

### 2.5. Preparation of Sample Solutions

Approximately 4.0 g of *Dimocarpus longan* leaf powder was weighed and put into a dry Erlenmeyer flask with plug. The powder was blended with 20 mL of the extraction solvent (ethanol : hydrochloric acid, 9 : 1, v/v). The mixture was ultrasonicated at 80°C for 50 min. After ultrasonication, extracts were replenished with the extraction solvent, then shaked, and centrifuged at a high speed. The supernatant was collected and stored at 4°C. The sample solutions were filtered through 0.22 *μ*m membrane filters before injection.

## 3. Results and Discussion

### 3.1. Representative HPLC/UPLC Chromatograms

**Figure 3:**
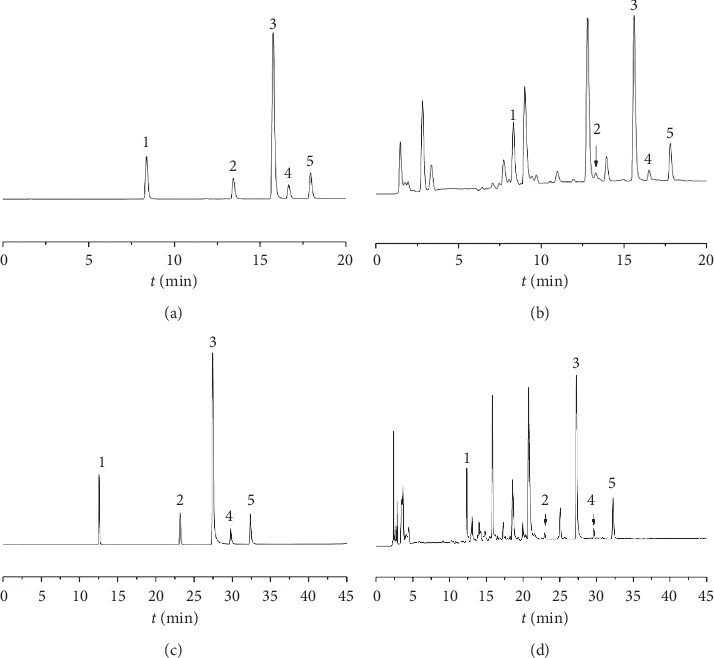
Chromatograms of the standard solutions and sample solutions. (1) Ethyl gallate. (2) Astragalin. (3) Quercetin. (4) Luteolin. (5) Kaempferol. (a) Mixed standard solution (UPLC); (b) sample solution (UPLC); (c) mixed standard solution (HPLC); (d) sample solution (HPLC).

The chromatograms of mixed standard solutions and sample solutions were taken for analysis under the aforementioned corresponding conditions. The results testified that separation of the 5 components in *Dimocarpus longan* leaves with the peaks freed from interference by adjacent peaks was good. Resolution values of the 5 components were greater than 1.5, and the chromatograms are shown in Figure 3.

### 3.2. Method Validation

#### 3.2.1. Calibration Curves

Working solution of mixtures of five standards was injected into the UPLC and HPLC system separately in a series of volumes. Analysis was proceeded in accordance with the corresponding chromatographic conditions mentioned in Section 2.3. The peak area (A) was fitted linearly with the mass of substance (*µ*g) for establishing calibration curves. The results showed that the correlation coefficients of all standards were at least 0.9996 that presented good linear relations in the test range. The evaluation results are shown in Table 1.

#### 3.2.2. Precision Test

The working solution of standards was injected 6 sequential times into the UPLC and HPLC systems, respectively, and the peak area of each component was recorded. In the UPLC system, the relative standard deviation (RSD) values of peak area of C_1_, C_2_, C_3_, C_4_, and C_5_ were 0.38%, 0.17%, 0.18%, 0.34%, and 0.23%, respectively, while in the HPLC system, 0.69%, 0.65%, 0.92%, 1.98%, and 1.05%, indicating that the precision of the instruments could be recognized precisely.

#### 3.2.3. Stability Test

Stability was investigated by analyzing the sample solutions at 0, 4, 8, 12, 18, and 24 h at room temperature and recording peak area of each component. The results in the UPLC system showed that the RSD value of peak area of C_1_, C_2_, C_3_, C_4_, and C_5_ was respectively 1.68%, 1.52%, 0.39%, 0.73%, and 1.06%. It indicated that the sample solutions were stable within 24 h.

#### 3.2.4. Repeatability Test

The repeatability was determined by analyzing the 6 sample solutions dividually in the UPLC system, which were prepared in parallel according to the method in Section 2.5. The peak area of each component was recorded to calculate the RSD value. It was found that the RSD value of mass of C_1_, C_2_, C_3_, C_4_, and C_5_ was 0.21%, 0.85%, 0.51%, 1.02%, and 0.93%, respectively. The repeatability of the preparation method of sample solution was proved to be credible according to the results.

#### 3.2.5. Sample Recovery Test

Recovery tests were performed to verify the accuracy of the method by adding the mixed standard solutions with known amount into the certain amount (2.0 g) of *Dimocarpus longan* leaf powder (9 portions). The mixtures of solutions and powders were extracted under the condition in Section 2.5 and analyzed by the UPLC system. The recoveries of the 5 components were in the range 93.28%–103.74%, suggesting that the analysis methods were practicable.

### 3.3. Determination of Relative Correction Factors

#### 3.3.1. Multipoint Correction

In the linear range, the detector response is directly proportional to the mass (or concentration) of the substance. By establishing the RCF_s_ between the internal reference and the other components, the quantities of the components can be directly calculated in practical application [16, 17]. In this study, C_3_ was chosen as an internal reference. In linear range, the RCF_s_ of C_1_, C_2_, C_3_, and C_4_ which was on different mass points were calculated according to formula (4), and the results are listed in Table 2. In general, if the components under test were similar in structure, the closer the maximum ultraviolet absorption wavelength they had, the closer to 1 the ƒ_s*/i*_ value would be, the smaller the error would appear in practice, and the more feasible the application of QAMS method would be [18, 19].

ƒ_s*/i*_ value of C_3_, C_5_, and C_4_ were all close to 1 since the only structural difference of them is the hydroxyl substitution position. Compared with C_5_, there is one more glucose group in C_2_ (ƒ_s*/i*_ ≈ 0.5), which with less content in leaves of *Dimocarpus longan* turns out to be less than accurate relative in quantitative analysis by using the QAMS method. Besides, the relative inferior accuracy of peak location of C_1_ (ƒ_s*/i*_ ≈ 0.4) may be related to the large difference with internal reference in structure.

(4) $$\begin{array}{c} f_{\text{s}/i}=\frac{f_{\text{s}}}{f_{i}}=\frac{m_{\text{s}}A_{i}}{m_{i}A_{\text{s}}}, \end{array}$$

where *m*_s_ is the mass of the internal standard, *A*_s_ is the peak area of internal standard, *m*_*i*_ is the mass of the remaining components to be measured, and *A*_*i*_ is the peak area of the remaining components to be measured.

#### 3.3.2. Slope Correction Method

In he regression equation, the intercept is usually caused by system error. The slope correction method corrects the whole mean deviation caused by special point deviation in the multipoint correction method by means of ignoring error. Based on it, ƒ_s*/i*_ can be calculated directly in terms of the ratio of intercepts, that is, formula (5). Formula (6) derived from formula (5) can be used to quickly calculate the mass of each component [20]. The calculated results showed that within the linear range, *f*_C_3_/C_1__=0.400, *f*_C_3_/C_2__=0.493, *f*_C_3_/C_4__=0.820, and *f*_C_3_/C_5__=0.913 in the UPLC system; *f*_C_3_/C_1__=0.379, *f*_C_3_/C_2__=0.479, *f*_C_3_/C_4__=0.812, and *f*_C_3_/C_5__=0.909 in the HPLC system. The results of the slope correction method were similar to that of the multipoint correction method.

(5) $$\begin{array}{c} f_{\text{s}/i}=\frac{k_{i}}{k_{\text{s}}}, \end{array}$$

(6) $$\begin{array}{c} m_{i}=\frac{A_{i}}{k_{\text{s}}\cdot f_{\text{s}/i}}, \end{array}$$

where *k*_s_ is the slope of the internal standard and *k*_*i*_ is the slope of other components.

### 3.4. Durability Evaluation of RCFs

#### 3.4.1. Repeatability

To verify the repeatability of the RCFs, 6 sample solutions prepared in parallel from the same sample were analyzed. According to multipoint correction, the RSD value of *f*_C_3_/C_1__, *f*_C_3_/C_2__, *f*_C_3_/C_4__, and *f*_C_3_/C_5__ in the UPLC system was figured out to be 0.19%, 0.71%, 0.22%, and 0.47%, respectively, while in the HPLC system, it was 0.27%, 0.30%, 1.77%, and 0.42%, respectively. Repeatability of RCFs turned out satisfactory based on the results above.

#### 3.4.2. Robustness

In order to investigate the robustness of RCFs in different chromatographic conditions, including instruments, columns, flow rates, and column temperatures, the mixed standard solution was injected and analyzed. The results showed that the RCFs of each component were not significantly different (RSD < 2.28%) under the above influencing factors, as shown in Table 3.

### 3.5. Identification of Chromatographic Peaks

The relative retention time of the components under test in different chromatographic systems was investigated so as to identify the chromatographic peaks of the components when C_3_ was only used as standard reference. The results shown in Table 4 indicate that relative retention time is accurate for peaks location and can be used as a parameter for identification of chromatographic peaks.

### 3.6. Comparison of the QAMS Method with External Standard Method

**Figure 4:**
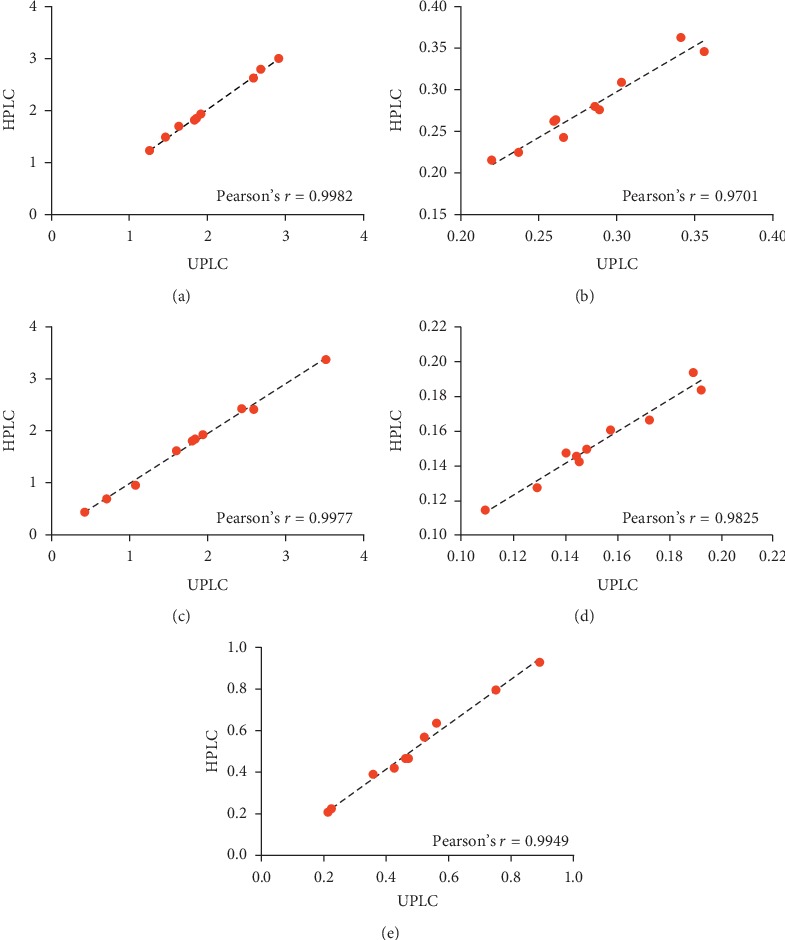
The similarity of detection results of different compounds by different chromatographic systems. (a) C_1_, (b) C_2_, (c) C_3_, (d) C_4_, and (e) C_5_.

At present, the external standard method (ESM) has become one of the most effective methods in the fields of multicomponent quantitative and qualitative analysis [21]. To evaluate the feasibility of the QAMS method, leaves of *Dimocarpus longan* were collected from 10 regions in Guangxi province in China to prepare sample solutions. Two instruments (UPLC and HPLC instruments) were used for sample analysis. Then, the amount of each component in the sample was calculated by using ESM, multipoint correction method (QAMS1), and slope correction method (QAMS2), respectively. The results are shown in Tables 5 and 6 and Figure 4; it was found that the quantitative results of 5 components calculated by the external standard method had significant correlation in different instruments and Pearson's coefficient (r) was at least 0.970. In addition, the results obtained by the QAMS method significantly correlated with the results of ESM, indicating that the different chromatographic systems and the method applied in this study were reasonable and feasible for the determination of the amounts of C_1_, C_2_, C_3_, C_4_, and C_5_ in *Dimocarpus longan* leaves.

## 4. Conclusion

The accurate identification of chromatographic peaks is a key part of the QAMS method. At present, relative retention time or retention time difference is commonly used to locate chromatographic peaks in most studies [22]. However, in most cases, positioning by the retention time difference is not effective and the data fluctuate severely [23]. Therefore, relative retention time is more widely used for peak positioning in the QAMS method. In this study, the relative retention time of the components was investigated in different chromatographic systems. The results showed that, in different chromatographic systems, the relative retention times of the components except C_1_ were all less than 1.04% with a negligible deviation, which could be used as the peak location parameters. But there are also studies that suggest the premise of applying relative retention time for positioning is to use chromatographic columns with the same filler and similar chromatographic behavior.

If the relative correction method is not effective, methods such as linear regression and trailing control can be adopted [18, 24, 25], or a little amount of standard reference can be used to qualitatively and accurately locate the peaks, and then the mass fraction of components can be calculated by using the RCFs [26].

In order to verify the rationality and feasibility of the application of the QAMS method for quantitating the 5 components in the leaves of *Dimocarpus longan*, methodological investigations including system adaptability test and durability evaluation of RCFs were carried out under different chromatographic systems. The results with good reproducibility were acquired in different conditions such as chromatography systems, columns, flow rate, and column temperature.

This study verified the universality of RCFs evaluated by the QAMS method in different chromatographic systems. In addition, the QAMS method was applied for determination of the content of *Dimocarpus longan* leaves for the first time, which laid the foundation for establishment of multi-index quality control of *Dimocarpus longan* leaves and also provided an alternative method in the absence of standard reference or instruments.

## Figures and Tables

**Table 1:** Results of linear relationships for the 5 components.

Component	UPLC			HPLC		
	Calibration curve	*r*	Linear range (*µ*g)	Calibration curve	*r*	Linear range (*µ*g)
C_1_	*Y* = 7794.28*x* − 16.64	0.99988	0.0490∼0.245	*Y* = 1354.52*x* + 17.42	0.99985	0.490∼2.450
C_2_	*Y* = 9618.87*x* − 13.22	0.99972	0.0216∼0.108	*Y* = 1713.83*x* + 4.38	0.99981	0.216∼1.080
C_3_	*Y* = 19503.76*x* − 50.67	0.99992	0.0832∼0.416	*Y* = 3574.23*x* + 80.52	0.99992	0.832∼4.160
C_4_	*Y* = 16010.58*x* − 7.42	0.99983	0.00920∼0.0460	*Y* = 2903.01*x* − 3.15	0.99966	0.0920∼0.460
C_5_	*Y* = 17806.81*x* − 15.31	0.99976	0.0144∼0.0722	*Y* = 3249.11*x* − 16.06	0.99964	0.144∼0.722

**Table 2:** Correction ƒ_s*/i*_ of 5 constituents.

Chromatographic system	Injection volume (*µ*L)	RCF_s_			
		*f*_C_3_/C_1__	*f*_C_3_/C_1__	*f*_C_3_/C_1__	*f*_C_3_/C_1__
UPLC	0.2	0.395	0.477	0.805	0.886
	0.4	0.397	0.485	0.813	0.900
	0.5	0.398	0.487	0.815	0.903
	0.6	0.398	0.490	0.816	0.907
	0.8	0.398	0.489	0.814	0.906
	1.0	0.399	0.490	0.818	0.908
Means		0.397	0.486	0.813	0.902
RSD (%)		0.34	0.96	0.51	0.82

HPLC	2	0.379	0.472	0.781	0.894
	4	0.379	0.476	0.797	0.882
	5	0.379	0.477	0.800	0.887
	6	0.379	0.477	0.802	0.891
	8	0.379	0.478	0.804	0.895
	10	0.379	0.478	0.806	0.898
Means		0.379	0.476	0.798	0.891
RSD (%)		0.02	0.39	1.02	0.62

**Table 3:** Robustness test of ƒ_s*/i*_.

Influencing factors		RCFs			
		*f*_C_3_/C_1__	*f*_C_3_/C_2__	*f*_C_3_/C_4__	*f*_C_3_/C_5__
UPLC	Column: Waters	0.397	0.479	0.817	0.902
	Column: Thermo	0.398	0.479	0.824	0.901
	Flow rate: 0.2 ml/min	0.397	0.481	0.812	0.897
	Flow rate: 0.3 ml/min	0.391	0.479	0.803	0.889
	Column temperature: 25°C	0.393	0.487	0.808	0.925
	Column temperature: 30°C	0.396	0.481	0.809	0.895
	Column temperature: 35°C	0.399	0.482	0.818	0.901
HPLC	Column: Phenomenex	0.376	0.471	0.785	0.879
	Column: Agilent	0.377	0.471	0.775	0.893
RSD (%)		2.28	1.06	2.07	1.48

**Table 4:** Relative retention time of 5 constituents.

Chromatographic systems		Relative retention time			
		*R*_t C_1_/C_3__	*R*_t C_2_/C_3__	*R*_t C_4_/C_3__	*R*_t C_5_/C_3__
UPLC	Column: Waters	0.541	0.828	1.062	1.179
	Column: Thermo	0.542	0.828	1.062	1.179
HPLC	Column: Phenomenex	0.495	0.844	1.086	1.180
	Column: Agilent	0.493	0.834	1.070	1.174
Means		0.518	0.833	1.070	1.178
RSD (%)		5.33	0.93	1.04	0.22

**Table 5:** Determination of 5 constituents using different instruments (mg/g, *n* = 3).

Regions	C_1_		C_2_		C_3_		C_4_		C_5_	
	UPLC	HPLC	UPLC	HPLC	UPLC	HPLC	UPLC	HPLC	UPLC	HPLC
Beihai	1.918	1.940	0.356	0.346	1.073	0.955	0.144	0.146	0.225	0.229
Qinzhou	2.679	2.796	0.341	0.363	2.438	2.425	0.157	0.161	0.561	0.639
Wuzhou	1.828	1.824	0.237	0.225	1.842	1.835	0.148	0.150	0.460	0.469
Yulin	1.631	1.698	0.261	0.264	1.799	1.799	0.140	0.148	0.425	0.421
Nanning	2.587	2.617	0.289	0.276	1.601	1.613	0.172	0.167	0.471	0.469
Chongzuo	1.850	1.827	0.266	0.243	0.426	0.443	0.109	0.115	0.213	0.210
Liuzhou	1.259	1.234	0.303	0.309	3.521	3.372	0.145	0.143	0.892	0.931
Guigang	1.880	1.882	0.220	0.216	2.589	2.410	0.189	0.194	0.751	0.798
Hezhou	2.915	3.002	0.260	0.262	1.936	1.918	0.192	0.184	0.522	0.571
Fangchenggang	1.462	1.490	0.286	0.280	0.699	0.685	0.129	0.128	0.358	0.392
Pearson's coefficient (r)	0.998^*∗∗*^		0.970^*∗∗*^		0.998^*∗∗*^		0.982^*∗∗*^		0.995^*∗∗*^	

^*∗∗*^At level 0.01 (two-tailed), the correlation was significant.

**Table 6:** Determination of 5 constituents by different quantitative methods (mg/g, *n* = 3).

Regions	C_1_			C_2_			C_4_			C_5_			C_3_
	ESM	QAMS1	QAMS2	ESM	QAMS1	QAMS2	ESM	QAMS1	QAMS2	ESM	QAMS1	QAMS2	ESM
Beihai	1.918	1.955	1.895	0.356	0.356	0.343	0.144	0.144	0.140	0.225	0.225	0.216	1.073
Qinzhou	2.679	2.702	2.656	0.341	0.336	0.327	0.157	0.155	0.153	0.561	0.566	0.553	2.438
Wuzhou	1.828	1.843	1.805	0.237	0.229	0.223	0.148	0.146	0.143	0.460	0.464	0.452	1.842
Yulin	1.631	1.643	1.609	0.261	0.255	0.247	0.140	0.139	0.136	0.425	0.428	0.416	1.799
Nanning	2.587	2.623	2.564	0.289	0.284	0.275	0.172	0.172	0.168	0.471	0.476	0.463	1.601
Chongzuo	1.850	1.959	1.827	0.266	0.272	0.252	0.109	0.112	0.104	0.213	0.221	0.205	0.426
Liuzhou	1.259	1.254	1.237	0.303	0.295	0.289	0.145	0.143	0.141	0.892	0.897	0.880	3.521
Guigang	1.880	1.888	1.857	0.220	0.212	0.207	0.189	0.188	0.184	0.751	0.759	0.742	2.589
Hezhou	2.915	2.950	2.891	0.260	0.253	0.247	0.192	0.192	0.188	0.522	0.527	0.514	1.936
Fangchenggang	1.462	1.505	1.439	0.286	0.287	0.273	0.129	0.130	0.124	0.358	0.368	0.349	0.699
Pearson's coefficient (r)		0.998^*∗∗*^	1.000^*∗∗*^		0.995^*∗∗*^	1.000^*∗∗*^		0.998^*∗∗*^	1.000^*∗∗*^		1.000^*∗∗*^	1.000^*∗∗*^	

^*∗∗*^At level 0.01 (two-tailed), the correlation was significant.

## Data Availability

The data used to support the findings of this study are available from the corresponding author upon request.
